# Secondary Progressive and Relapsing Remitting Multiple Sclerosis Leads to Motor-Related Decreased Anatomical Connectivity

**DOI:** 10.1371/journal.pone.0095540

**Published:** 2014-04-18

**Authors:** Mark Lyksborg, Hartwig R. Siebner, Per S. Sørensen, Morten Blinkenberg, Geoff J. M. Parker, Anne-Marie Dogonowski, Ellen Garde, Rasmus Larsen, Tim B. Dyrby

**Affiliations:** 1 Danish Research Centre for Magnetic Resonance, Copenhagen University Hospital Hvidovre, Hvidovre, Denmark; 2 Department of Applied Mathematics and Computer Science, DTU Compute, Technical University of Denmark, Kongens Lyngby, Denmark; 3 Danish Multiple Sclerosis Center, Copenhagen University Hospital Rigshospitalet, Copenhagen, Denmark; 4 Centre for Imaging Sciences, Biomedical Imaging Institute, University Of Manchester, United Kingdom & Bioxydyn Limited, Manchester, United Kingdom; Centre Hospitalier Universitaire Vaudois Lausanne - CHUV, UNIL, Switzerland

## Abstract

Multiple sclerosis (MS) damages central white matter pathways which has considerable impact on disease-related disability. To identify disease-related alterations in anatomical connectivity, 34 patients (19 with relapsing remitting MS (RR-MS), 15 with secondary progressive MS (SP-MS) and 20 healthy subjects underwent diffusion magnetic resonance imaging (dMRI) of the brain. Based on the dMRI, anatomical connectivity mapping (ACM) yielded a voxel-based metric reflecting the connectivity shared between each individual voxel and all other brain voxels. To avoid biases caused by inter-individual brain-shape differences, they were estimated in a spatially normalized space. Voxel-based statistical analyses using ACM were compared with analyses based on the localized microstructural indices of fractional anisotropy (FA). In both RR-MS and SP-MS patients, considerable portions of the motor-related white matter revealed decreases in ACM and FA when compared with healthy subjects. Patients with SP-MS exhibited reduced ACM values relative to RR-MS in the motor-related tracts, whereas there were no consistent decreases in FA between SP-MS and RR-MS patients. Regional ACM statistics exhibited moderate correlation with clinical disability as reflected by the expanded disability status scale (EDSS). The correlation between these statistics and EDSS was either similar to or stronger than the correlation between FA statistics and the EDSS. Together, the results reveal an improved relationship between ACM, the clinical phenotype, and impairment. This highlights the potential of the ACM connectivity indices to be used as a marker which can identify disease related-alterations due to MS which may not be seen using localized microstructural indices.

## Introduction

Multiple sclerosis (MS) is a disease with an inflammatory, demyelinating and neurodegenerative disease course. It diffusely affects the structural and functional integrity of white matter (WM) tracts, contributing to disease-related disability. Regional structural abnormalities can be readily detected with magnetic resonance imaging (MRI) which explains the pivotal role of conventional MRI techniques for early diagnosis of MS [1]. However, the WM lesions seen on T2-weighted MRI only weakly correlate with disease-related disability [2]–[3]. The poor relation between clinical disability and lesion loads as revealed by T2-weighted MRI has motivated the search for other MRI markers which may reflect more closely the disease-related structural changes leading to disability.

Diffusion magnetic resonance imaging (dMRI) is an imaging modality sensitive to the mobility of water molecules. It is sensitive to disease-induced changes in microstructure because the diffusion process of water is determined by the microstructural boundaries of the local brain tissue environment. To characterize the diffusion properties within a voxel and to estimate the most probable axonal fiber direction, diffusion tensor imaging (DTI) can be used [4]. The DTI model enables the estimation of simple scalar diffusion indices such as the mean diffusivity (MD), fractional anisotropy (FA) [5] and many others. The indices have been successfully applied to study microstructural abnormalities in patients with MS, showing reduced FA (increased MD) in MS lesions and normal appearing white matter (NAWM) [6]–[8], presumably reflecting axonal damage [9]. Further, changes in the DTI indices have been linked to disease-related disability [10] and to specific clinical phenotypes [11].

While DTI indices reflect localized microstructural properties of brain tissue, other indices such as anatomical connectivity mapping (ACM) are able to give a more complex description of the health state of the WM network. ACM does this by estimating a connectivity index reflecting the strength of connectivity a voxel has with the rest of the brain. Since MS is a very heterogeneous disease and each voxel of the ACM may be sensitive to microstructural damage at a distant site, we believed it may be able to supply disease contrast not seen with localized DTI indices. The voxel-specific whole-brain anatomical connectivity indices of ACM are estimated by initiating probabilistic streamlines from all voxels within a whole-brain mask and counting the number of streamlines passing through each voxel within the mask. The ACM approach was first proposed by [12] and has recently been applied to identify brain structures showing reduced whole-brain anatomical connectivity in patients with Alzheimer's disease [13] and in patients with a relapsing remitting (RR) course of MS [14]. The latter study revealed reduced anatomical connectivity in the thalamus and caudate nucleus of an MS group relative to a healthy group. In addition, ACM values in the corpus callosum, right hippocampus and cerebellum showed a positive linear relationship with individual scores of the Paced-Auditory-Serial-Addition-Test (PASAT) [15], suggesting that the anatomical connectedness of these structures might be particularly relevant to measuring cognitive function in MS.

Here we apply ACM to study the disruption of anatomical connectivity in relapsing remitting MS (RR-MS) and secondary progressive MS (SP-MS). Unlike previous works we minimized the influence of inter-subject anatomical differences by estimating ACM in a common stereotactic space. Given the diffuse pathology of MS, we hypothesized ACM to reveal widespread reductions in motor-related WM compared with healthy subjects and that these reductions will differ depending on the clinical form of MS. The principal reasoning behind the motor-related hypotheses is that MS patients are expected to experience motor disabilities. The motor-related reduction should be apparent when comparing patients to healthy subjects and RR-MS patients to SP-MS patients. The between MS group connectivity reduction is hypothesized since the SP-MS patients of our study exhibit an increased motor disability compared to RR-MS patients, as measured by a higher extended disability status scale (EDSS) score [16].

## Materials and Methods

### 2.1 Subjects

Thirty-four patients suffering from MS fulfilling the revised McDonald criteria [17] participated in this study (Table 1) of whom 19 patients had been diagnosed with the RR-MS disease course, while 15 patients had been diagnosed with the SP-MS disease course. Patients were recruited from the Danish Multiple Sclerosis Center (-based at Rigshospitalet, Copenhagen, Denmark). We only included clinically stable patients who had not experienced a relapse in the three months preceding the MRI measurement were included in the study. MS patients with neurological co-morbidity and with signs of depression or other psychiatric disorders were excluded from the study. All patients were neurologically examined and clinical disability was rated using the EDSS score [16]. The patients were treated with disease-modifying drugs (interferon-beta, glatiramer acetate, natalizumab). Twenty healthy subjects without a history of neurological or psychiatric disease were included as control subjects. The healthy subjects were age matched with the group of MS patients but not with their gender distributions.

**Table 1:** Clinical and demographics of the study population.

Characteristics	Healthy	RR-MS	SP-MS	p-value	p-value	p-value
	n = 20	n = 19	n = 15	Healthy-RR	Healthy-SP	SP-RR
Age (years)	44.60±9.87	39.45±8.98	49.34±11.31	0.10	0.19	0.01
	(25,68)	(24,56)	(29,64)			
Gender	13m/7f	6m/13f	6m/9f	NA	NA	NA
EDSS	NA	3.05±1.4	5.36±1.14	NA	NA	<0.001
		(0, 4.5)	(3.5,7)			
Lesion loads (ml.)	NA	21.78±15.93	32.70±19.41	NA	NA	0.05
		(3.03, 64.65)	(3.69, 60.66)			
Duration (years)	NA	10.63±5.90	21.20±11.12	NA	NA	<0.01
		(3,27)	(6,43)			

**Legend:** Lists the mean ± standard deviations, the range of the population characteristics (in parenthesis) and the significance values (the p-value) of group-wise comparisons based on two-sample, two-tailed t-test. The lesion loads are un-normalized loads. The entry, NA means not applicable.

#### 2.1.1 Ethics Statement

The study was approved by the Scientific Ethical Committee of the municipalities of Copenhagen and Frederiksberg (protocol no. KF01 – 131/03 with addendum) and all subjects gave written informed consent.

### 2.2. Magnetic resonance imaging

All MRI data were acquired on a Siemens TRIO 3 tesla scanner using an eight-channel surface head-coil (In vivo, FL, USA). The structural MRI protocol included a three-dimensional T1-weighted image using a magnetization prepared rapid gradient echo (MPRAGE) sequence with a repetition time (TR) of 11400 ms, echo time (TE) of 2.32 ms, flip angle (FLA) of 9° and a matrix of 182×218×182, resulting in a 1 mm^3^ isotopic resolution. Also acquired was a three-dimensional T2-weighted image (T2) with a TR of 3000 ms, TE of 354 ms, FLA = 180° and a matrix of 196×256×192, resulting in a 1.1×1.1×1.1 mm^3^ isotropic resolution and a three-dimensional T2-weighted fluid attenuation inversion recovery (FLAIR) image using the sequence parameters, TR = 6000 ms, TE = 353 ms, FLA = 180° and a matrix of 220×256×192 (1.1×1.1×1.1 mm^3^ resolution).

Diffusion MRI (dMRI) was acquired using a twice-refocused spin echo sequence [18] (TR = 8200 ms, TE  = 100 ms, in-plane matrix = 96×96; 61 slices) resulting in 2.3×2.3×2.3 mm^3^ isotropic voxel resolution. For each subject, ten non-diffusion weighted images (b0) and 61 diffusion weighted images (DWIs) were acquired using a b-factor of 1200 s/mm^2^. In addition, a field map was obtained used a double gradient echo sequence (TR = 479 ms, TE_short_ = 5.19 ms, TE_long_ = 7.65 ms, FLA = 60°, matrix = 128×128; 47 slices) resulting in a voxel resolution of 2×2×3 mm^3^.

### 2.3. Preprocessing

The DWIs were simultaneously corrected for eddy currents and motion by co-registering them with the first of the ten b = 0 images using a 12 parameter affine transformation [19]. To correct for susceptibility artefacts, a voxel displacement map was estimated based on the individual field maps and the field map correction approach, available through SPM8 (http://www.fil.ion.ucl.ac.uk/spm/) [20]. The voxel displacement map and the affine model displacements were combined and the DWIs re-sliced into the space of the first b0 image using tri-cubic interpolation. After re-slicing the DWIs, the gradient directions of the DWIs were updated by applying the rotational part of the affine model to the gradient directions [21].

Based on T2, FLAIR and MPRAGE modalities WM lesions were segmented using an automated segmentation method described by [22]. A radiographer manually adjusted the segmented lesions, resulting in a binary lesion mask for each MS subject. The FLAIR image was co-registered with the first b0 image of the dMRI and both the FLAIR and the lesion mask re-sliced into the space of the b0 image, using tri-cubic and nearest-neighbour interpolation for the respective images.

### 2.4 Anatomical Connectivity Mapping (ACM)

#### 2.4.1 Fiber reconstruction: The multi-tensor

The multi-tensor model by [23] was chosen as the multi-fiber model and the parameters of the model were fitted using a Levenberg-Marquardt optimization available through the Camino software (http://cmic.cs.ucl.ac.uk/camino/) [24]. The maximum number of tensors was fixed to two per voxel, as the findings reported in [23] suggest this to be the maximum number of resolvable fibers using a b-factor near 1000 s/mm^2^. To determine the number of fibers in a voxel, we used the classification approach proposed by [25], also from Camino.

#### 2.4.2 Spatial normalization of the multi-tensors

To ensure ACMs with cross-subject comparability, it is important to prevent cross-subject brain shape variability from biasing the connectivity values of the ACM. To avoid this, the tensor models were spatially normalized into a common space [21], [26] prior to calculating the ACM. This strategy differs from calculating the ACM in native space and then normalizing the ACM, as was done in previous ACM studies [13]–[14]. The optimal spatial normalization was found by matching the FA map of each subject with the FMRIB58_FA atlas (58 healthy subjects) [27], using the FSL image registration routines flirt and fnirt (http://www.fmrib.ox.ac.uk/fsl/) [28]. The routines estimate the spatial normalization as a concatenation of an affine deformation model (flirt) and a non-rigid B-spline deformation model (fnirt). The affine model takes care of spatially normalizing the coarser structures of the brain while the more flexible B-spline model takes care of spatially normalizing with respect to the finer structures of the brain. The cost function criteria used for estimating the spatial normalization and matching FA maps was the sums of square difference criteria. To prevent severe MS pathology from influencing the spatial normalization, the option of cost function masking was enabled, using lesion masks to ensure that the spatial normalization is derived solely from tissue outside of lesions. The result of the spatial normalization was used to transform the FA images and the multi-tensors of the subjects. To ensure proper orientation of the tensors after spatial normalization, the preservation of principal direction (PPD) algorithm [21] was used to re-orient each single tensor of the multi-tensor. The FA and multi-tensor volumes were both re-sliced to 2×2×2 mm^3^ isotropic resolutions matching the FMRIB58_FA atlas.

#### 2.4.3 Probabilistic whole-brain tractography

The values of an ACM reflect the connectivity/dis-connectivity that each individual voxel has with the rest of the brain. The ACM is estimated by repeating probabilistic tractography for every seed voxel of the brain and counting the number of probabilistic streamlines passing through each individual voxel [12]. Besides the probabilistic tractography method, the ACM estimation also requires the specification of a binary brain mask identifying the seed voxels.

The brain mask was derived from the FMRIB58_FA atlas by identifying voxels with FA≤0 as background and the rest as voxels belonging to the brain mask. This definition is inherited directly from the FMRIB58_FA atlas documentation. This whole-brain mask is used both as a seed mask and as a streamline stopping criteria. We used the PICo probabilistic tractography method by [29] as implemented in Camino [24], to propagate streamlines. For this purpose the fiber orientation distribution function (fODF) of each voxel was simulated using the Bingham distribution under the assumption of a signal-to-noise ratio (SNR) set to 16. Probabilistic tracking is performed using, an interpolated tracking approach with a tracking step size of 1/10 the voxel size and using a combination of two streamline termination criteria. A streamline is terminated when it leaves the interior of the brain mask and if it turns more than 180 degrees across the extent of a voxel. The ladder criterion prevents a streamline from doubling back on itself.

#### 2.4.4 Number of streamlines

To minimize the uncertainty associated with voxel-wise connectivity estimates of an ACM it is important to choose a sufficiently large number of streamlines. We therefore estimated the average uncertainty of each ACM voxel as a function of the number of streamlines in five healthy subjects. Individual subject ACMs were estimated five times for a fixed number of streamlines (i.e., 10, 50, 100, 150, 300, 500, 700 streamlines per seed voxels) and the voxel-wise coefficient of variation (CV) was calculated [30]. For a given number of streamlines, the average CV across all voxels within the seed mask reflects the estimation uncertainty (the precision of an ACM estimate). The average CV function is inversely proportional to the average SNR, and for the sake of completeness the result section will supply both.

### 2.5 Lesion probability map

The estimation of a lesion probability map was done by deforming and resampling all lesion masks towards the common atlas space (FMRIB58_FA), using the spatial normalization previously described. This was followed by a voxel-wise summation of the spatially normalized lesion masks, each voxel quantifying the number of observed lesions divided by the total number of subjects.

### 2.6 Group-wise statistical analysis

#### 2.6.1 Between-group comparison

Regarding the voxel-based measures of whole-brain connectivity, we hypothesized that both, the group of RR-MS and SP-MS patients, would show increased connectivity relative to a group of healthy subjects. In addition, we expected that SP-MS patients (with a higher range of EDSS scores) would display reduced connectivity relative to RR-MS patients (with a lower range of EDSS scores). These hypotheses, commonly referred to as alternative hypotheses, were examined by testing the null hypotheses which assumes no differences between groups. The hypotheses were investigated using a general linear model (GLM), including group, age, gender and a voxel-based atrophy measure as independent covariates while using the ACM values as the dependent variable. The purpose of the voxel-based atrophy covariate is to adjust for potentially confounding effects that tissue atrophy may have on the spatially normalised ACM and FA. For instance, atrophy in the form of enlarged ventricles might introduce consistent errors in the spatial normalization process [31] between patient groups of varying pathology. To minimize such errors from impacting the statistical analysis the voxel-based atrophy covariate was used. To estimate the voxel-based atrophy covariate a WM mask was formed in atlas space using FMRIB58_FA>0. This mask was modulated with the determinant of the Jacobian of the deformation field that spatially normalizes from subject to atlas space. Each voxel of this modulated mask reflects the amount of tissue expansion/contraction experienced in a voxel. To remove the influence that cross subject brain size may have on the modulated Jacobian determinant, each of the modulated voxels were linearly scaled using the intra cranial volume (ICV). The ICV was calculated as the sum of WM, grey matter (GM) and cerebrospinal fluid (CSF) segmentations, estimated using the SIENAX [32] segmentation routine of FSL 4.0.

Prior to fitting voxel-wise GLMs, the ACM data were smoothed using a Gaussian kernel with a full-width half-maximum value of 4 mm. After fitting the GLMs, the GLM parameters were examined using one-tailed two sample t-tests with the assumption of unequal variance, testing for voxel-wise significant differences between the group parameters of the GLM. The same voxel-based GLM analyses were repeated using FA. The additional FA-based analyses enabled us to compare the outcome of analyses based on a measure reflecting localized microstructural tissue properties (FA) with analyses based on a measure reflecting whole-brain connectivity (ACM).

The corticopontine tracts and superior longitudinal fascicles of the left and right cerebral hemispheres were defined as tracts of interests. We chose the corticopontine tracts because they contain the major descending corticospinal and corticonuclear motor fibers and therefore, are critical to motor function [33]–[34] where motor function is known to be strongly affected by MS as measured by the EDSS score [16], [35]. Likewise, the superior longitudinal fascicule is closely related to motor function such as movement execution and imagery [36]. These motor-related tract regions have also been examined in previous DTI-related neuroimaging studies in MS and the involvement of these tracts in MS have been shown to be of relevance [11], [37]–[38]. For these tracts of interests, we applied a small volume correction (SVC) approach to account for the problem of multiple comparisons [39]–[41]. The tract of interest ROIs required for the SVC, were given by the expert delineations of the JHU-ICBM-DTI-81 atlas [42] which are in correspondence with the FMRIB58_FA atlas space. The ROI tracts were assumed to coincide with the subset of the JHU-ICBM-DTI-81 atlas delineations, consisting of the internal capsule, the cerebral peduncle, the corticospinal tract, the corona radiata and the bilateral superior longitudinal fasciculi. Within these ROIs, the SVC method of the software toolkit SPM8 was used to correct for multiple non-independent comparisons, yielding a family wise error (FWE) corrected statistical threshold. The statistical significance of this FWE was set to 0.05.

#### 2.6.2 Within-group correlation analyses

We expected that regional reduced anatomical connectivity in the motor-related tracts might be closely related to inter-individual variations in clinical disability. The analyses were conducted by estimating a regional median ACM statistic within the aforementioned ROIs, which were correlated with the EDSS scores using Pearson's correlation coefficient. For the sake of comparison, the linear correlation was also calculated for the median FA within the ROIs. These analyses were supplemented by GLM analyses using models adjusted for confounding covariates. We fit the GLM using EDSS as the outcome measure, and using the median statistic, age and gender as explanatory covariates. The fitting correlation of the GLM and its coefficient of determination r^2^ [30] were used as indicators of the disease-related information inferred from the ACM and FA respectively. To adjust for the multiple comparison problem of investigating correlations in several WM ROIs, the Boneferroni correction [30] was used. The Boneferroni significance was chosen such that the probability of observing a false positive was less than 5 percent.

White matter lesion load is often used as an imaging marker in MS treatment studies [43]. As a metric of reference we therefore estimated the individual skull normalized lesion loads which were correlated with inter-individual variations in the EDSS score. The skull normalization was estimated using the SIENAX routine of FSL 4.0 [32].

## Results

### 3.1 Number of streamlines

ACM estimation using probabilistic tractography with different numbers of streamlines per voxel revealed a decreasing SNR benefit as the number of streamlines increases (Table 2). SNR became stable in the range 300 to 700 streamlines, prompting us to choose 500 streamlines per seed voxel to yield asymptotically stabile ACM estimates. Depending on the desired average SNR, the ACM may be estimated with fewer streamlines.

**Table 2:** Uncertainty associated with ACMs for increasing number of streamlines.

Number of streamlines	10	50	150	300	500	700
Average CV	0.049	0.023	0.014	0.012	0.011	0.012
Average SNR	20.32	44.21	69.44	85.84	87.42	85.54

**Legend:** Shows the average voxel-wise coefficient of variation (CV) and the average signal to noise ratio (SNR) for repeated ACM estimation to investigate the effect of the number of streamlines. The average CV/SNR values are based on a CV/SNR volume of five healthy subjects where each of the volumes comes from a five times repeated estimation of the same ACM.

### 3.2 Reduced connectivity in patients compared to healthy subjects

**Figure 1:**
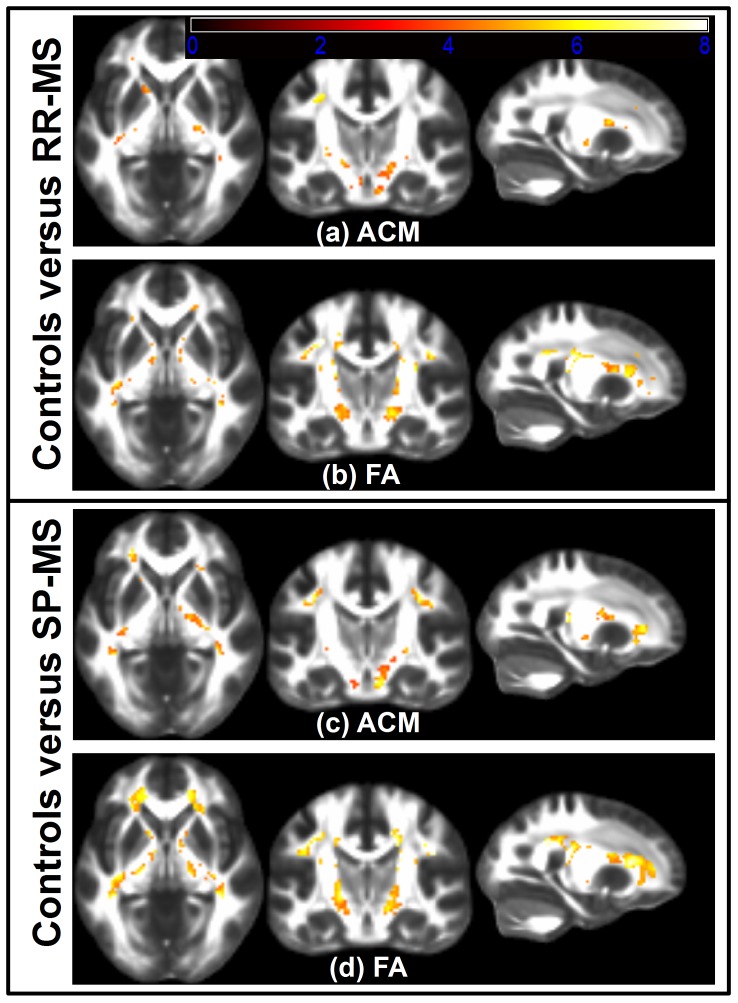
Voxelwise comparison between MS patients and healthy subjects. The axial, coronal and sagittal slices of (**a**)–(**d**) are shown in neurologic convention. Slices in (**a**) shows voxel-based t-tests, thresholded at the SVC significance level of 0.05, used to test if the ACMs of RR-MS patients are decreased relative to healthy subjects while (**b**) shows the corresponding, thresholded t-test based on FA. (**c**) and (**d**) shows the results of a similar comparison test between SP-MS patients and healthy subjects with the t-tests based on ACM depicted in (**c**) and the t-tests based on FA shown in (**d**). The background images shown are from the FMRIB58_FA atlas.

**Figure 2:**
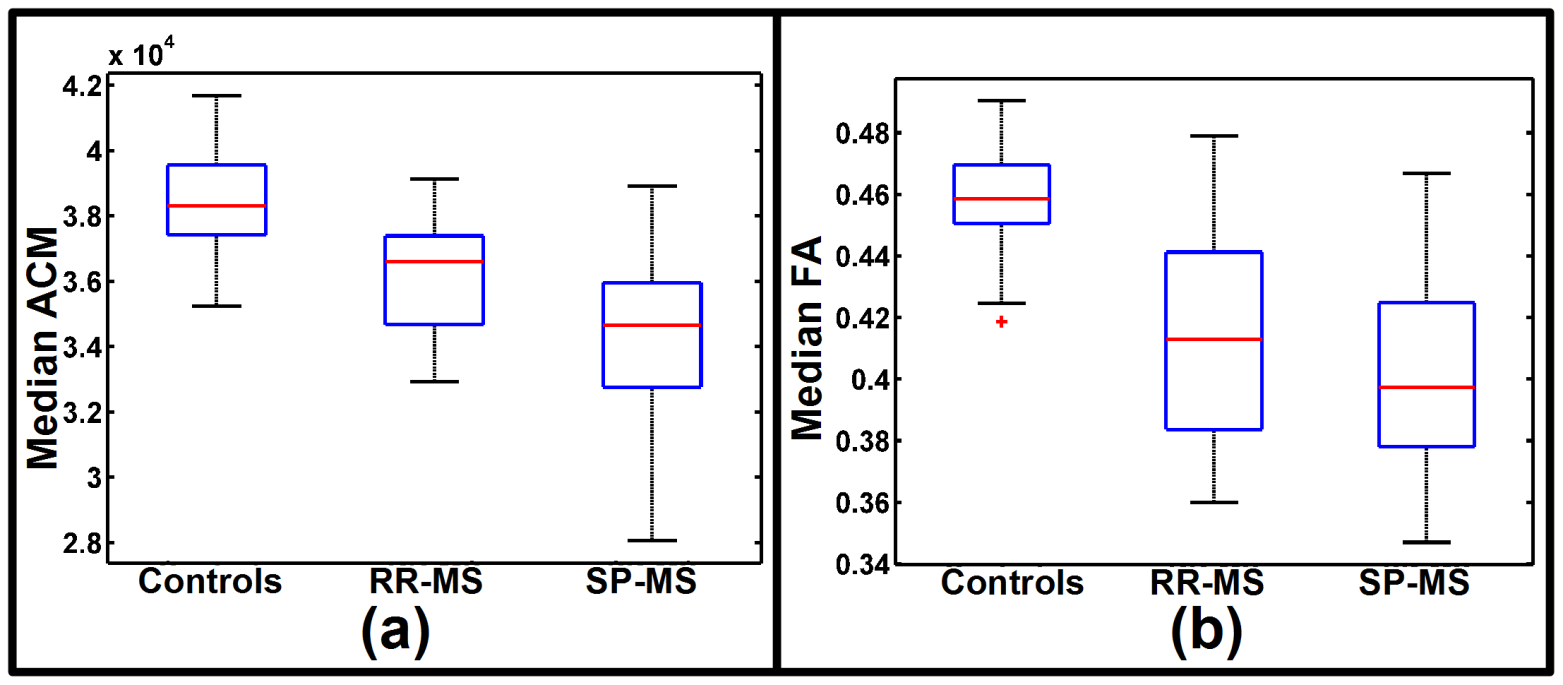
Group distributions of summary statistics from the bilateral motor-related tracts. The box plot in (**a**) shows the group distributions of the median ACM statistics estimated from voxels within a WM region of interest delineating the bilateral motor-related tracts. The group divisions are healthy subjects, RR-MS and SP-MS patients. The box plot in (**b**) shows the corresponding distributions of the median FA statistics.

Using the voxel-based GLM it was found that patients with RR-MS and SP-MS had widespread ACM reductions in all motor-related WM compared to healthy subjects as shown in Figure 1(a) and 1(c). These figures show the voxels surviving the SVC within the pre-defined WM ROIs. The widespread reductions in anatomical connectivity were also reflected by a significantly lower median ACM statistic within the RR-MS and SP-MS groups, compared with the median ACM of the healthy subjects (p-value<0.0001, one-tailed two-sample t-test; Figure 2(a)). While both groups displayed significantly decreased voxel-wise ACM values in the WM, there were overall more voxels showing reductions in SP-MS patients relative to patients suffering from RR-MS (Figure 1 (a) and 1(c)).

Patients with RR-MS and SP-MS also demonstrated widespread decreases in FA (Figures 1(b) and 1(d)), resulting in significant FWE-corrected voxels within the WM. Accordingly, the median FA statistics of the WM were significantly reduced in patients with RR-MS and SP-MS (p-value<0.0001, two-sample t-test; Figure 2(b)).

In summary, these results show that the pathology changes of both RR-MS and SP-MS are associated with widespread motor-related decreases in ACM and FA. They further suggest that based on summary statistics, FA and ACM appear equally informative about the clinical phenotypes.

**Figure 3:**
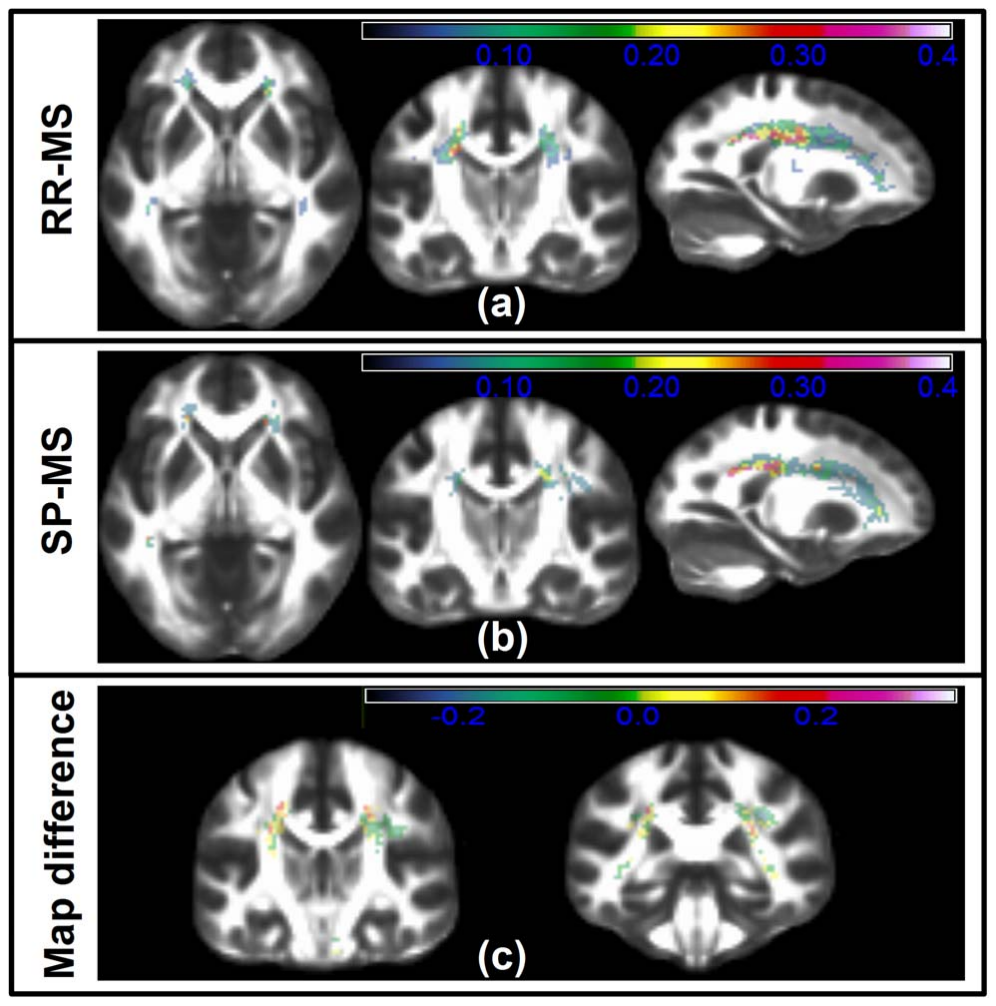
The lesion probabilities of MS phenotypes. (a), (b) and (c) show lesion probability maps of slices corresponding to those of Figure 1 and 4. The lesion probability map of RR-MS patients is shown in (**a**) while the corresponding map based on SP-MS patients are shown in (**b**). The overlaid color maps and color bars of (**a**), (**b**) indicates the lesion probability/frequency within the bilateral motor-related WM tracts. In (**c**) the difference the two phenotype maps are show equivalent to MAP_SP-MS_-MAP_RR-MS_. Thus a positive color map suggests a region where the SP-MS group more frequently contains a T2 lesion relative to the RR-MS patient group.

Comparing the findings of Figure 1 to Figure 3(a)–(b), we observed that the significant voxels found using ACM and FA have a moderate overlap with larger than zero lesion probability. This overlap was further quantified using the dice score [44], comparing the extent of the significant voxel regions with the extent of the lesion map. This score lies in the interval [0∶1], where 1 indicates a perfect overlap between the regions. Using the significant voxels based on ACM, the dice scores were 0.015 and 0.14 for the respective groups of RR-MS and SP-MS. Based on significant voxel regions derived from FA, the dice scores were 0.32 and 0.42. These results suggest an increased relation between severe focal pathology and FA relative to ACM. They also indicate that only a small proportion of the decreased FA/ACM voxels are directly related to structurally identified lesion positions. This was particularly the case when considering the ACM results.

### 3.3 Decreased connectivity of SP-MS relative to RR-MS

**Figure 4:**
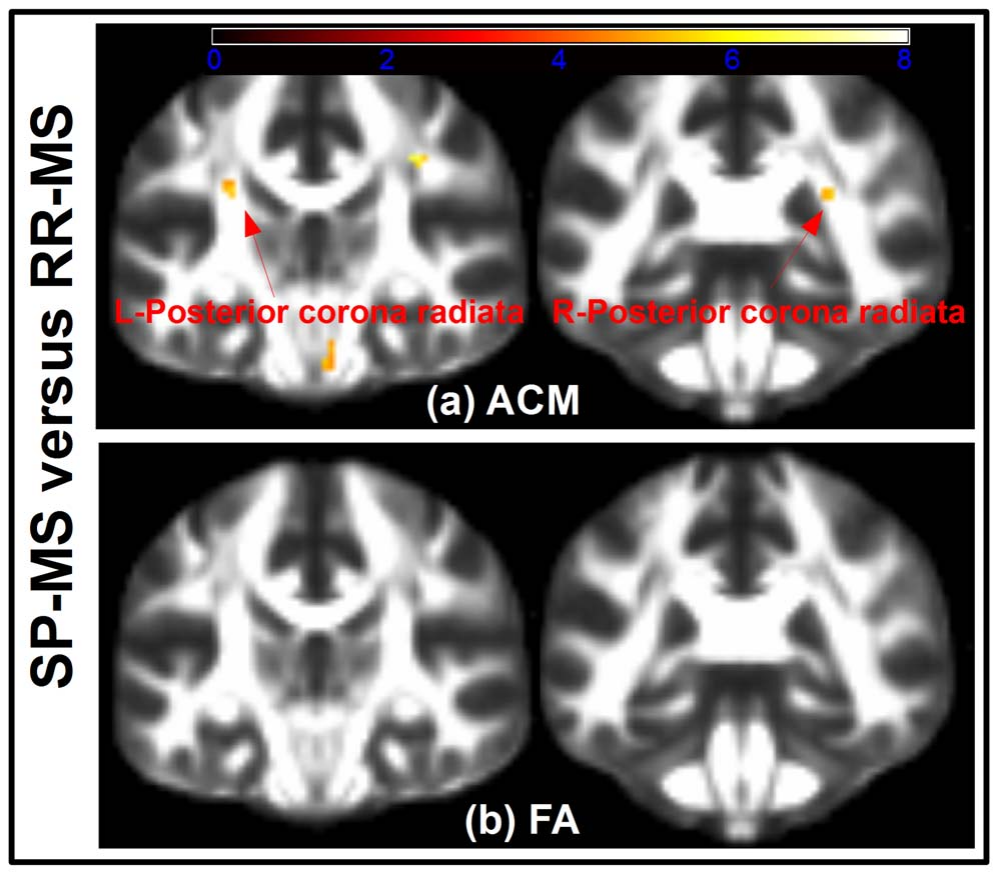
Voxelwise comparison between the phenotypes of RR-MS and SP-MS. (**a**) Shows t-test voxels, thresholded at SVC significance level of 0.05. The coronal slices show where the SP-MS patients have significantly reduced ACM compared to RR-MS patients. The voxel-based t-tests reveal significant reductions at the left posterior corona radiata (coronal slice 46, shown on the left) and the right posterior corona radiata (coronal slice 52, shown on the right). The embedded arrows points to the regions of significant voxels. Similarly, (**b**) shows the result of testing where the SP-MS patients have a decreased FA compared to the RR-MS patients and no significant voxels are found. The t-tests depicted in (**a**) and (**b**) have been overlaid the FMRIB58_FA atlas.

We found the median ACM statistics in the motor-related WM to be decreased in patients with SP-MS relative to patients with RR-MS (p = 0.013, two-sample t-test; Figure 2(a)). In contrast, median FA statistics were not significantly different between the two groups (p = 0.126, two-sample t-test; Figure 2(b)). Voxel-based GLM analysis revealed localized decreases of the ACM values in patients with SP-MS relative to RR-MS (Figure 4(a)). Within the aforementioned bilateral WM ROIs, we identified regions where the ACM values were significantly reduced in SP-MS patients relative to patients with RR-MS (Figure 4(a); Table 3). Table 3 lists the location of these spatially coherent significant voxels. A large number of FWE significant voxels were located in the left and right posterior corona radiata, the right corticospinal tract as well as the right superior longitudinal fasciculus. In contrast, the same voxel-based group comparisons based on FA revealed no FWE-corrected significant differences between SP-MS and RR-MS and for this reason the results are not reported. The significant FWE t-tests based on FA are depicted in Figure 4(b) where the lack of significant voxels underline the difference between ACM and FA based analyses. According to the t-test comparison in Table 1 there is a significant EDSS difference between the SP-MS and the RR-MS groups. A useful phenotype disease marker is expected to reflect this difference and Figure 4 shows that ACM can indeed reveal phenotype related differences that are not seen using FA. Relating this finding with the lesion probability difference map of Figure 3(c) we note that the difference map appears quite noisy. This makes it challenging to assess whether there are spatially consistent lesion differences between the two groups that could give rise to the consistent phenotype differences observed with ACM. We do observe a small trend of the negative and positive difference in certain brain regions. However a t-test comparing the voxel-wise proportions of SP-MS and RR-MS reveal no significant SVC differences between their lesion patterns. Given the large regional extent of the lesion difference map and the low number of significant ACM and FA voxels, a dice score overlap metric between the voxels and the region becomes un-informative (close to 0).

**Table 3:** Regions of significantly decreased ACM of SP-MS relative to RR-MS patients.

Region of interest	Nvox	P_FWE-peak_	MNI-coordinates (x, y, z) - in mm
R-superior longitudinal fasciculus	33	6•10^−6^	(41, 13, 31)
L-posterior corona radiate	20	1•10^−5^	(−27, −35, 25)
R-posterior corona radiate	16	3•10^−5^	(29, 59, 23)
R-corticospinal tract	12	1•10^−4^	(5, 23, 31)

**Legend:** The Region of interest column list the name of a WM ROI delineated in the JHU-ICBM-DTI-81 atlas with the letters R and L referring to a hemisphere. The second columns (Nvox) are the number of voxels in a region surviving the SVC statistical threshold of 0.05. The third column (P_FWE-peak) lists_ the significance of the largest t-test voxel of a region. The last column (MNI-coordinates) contains the spatial position of this voxel.

The difference between the FA and ACM analysis results is likely because the ACM indices are increasingly global in nature as opposed to FA. We believe this property in part overcomes analysis difficulties caused due to different MS subjects with similar symptoms but having spatially heterogeneous disease pathologies. The effect of such heterogeneous pathology pattern is observed by the non-significant and widespread differences between the RR-MS and SP-MS lesion maps in Figure 3(c).

### 3.4 Correlations between ACM and clinical disability

**Figure 5:**
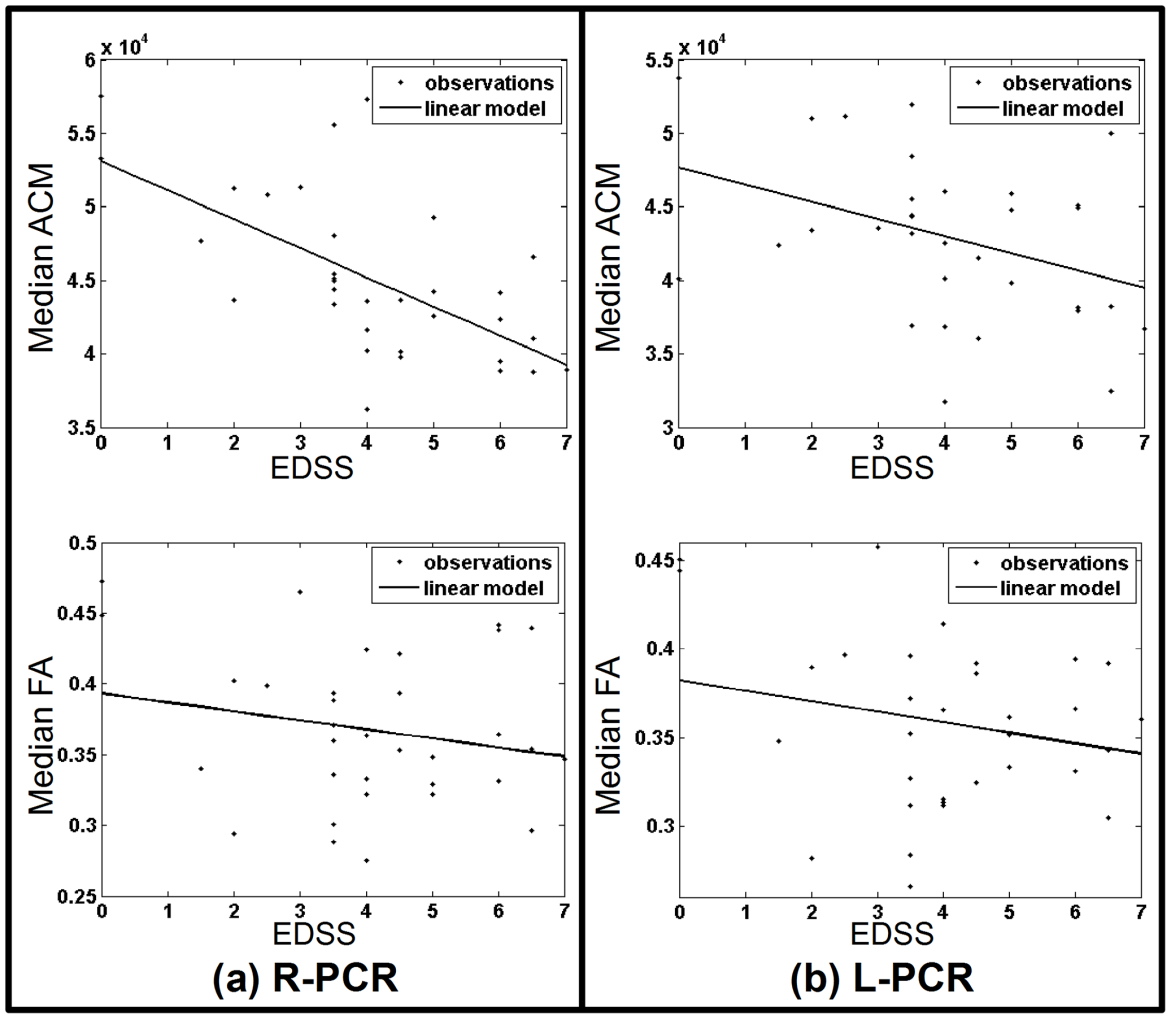
Disease score correlations. Shows the median ACM and the median FA plotted against EDSS disease score where the ACM and FA indices are sampled from a specific WM region of the JHU-ICBM-DTI-81 atlas. Subfigure (**a**) shows a cross subject plot of the median ACM and the median FA summary statistics obtained from the right posterior corona radiate (R-PCR). The data points are depicted with dots while a general linear model (GLM) fit is shown as a solid line. Similarly, (**b**) shows the median ACM and median FA plots based on the left posterior corona radiate (L-PCR).

The region-specific median ACM statistics within the motor-related WM tracts showed significant correlations with the individual EDSS scores (Table 4). Correlation based on the corresponding FA statistics generally did not result in the same significance (Table 4). Instead, using a GLM adjusted for age and gender effects resulted in significant EDSS correlations both for ACM and FA (Table 4). The magnitude of the correlations between the ACM and EDSS scores, as well as the amount of variance explained by the GLM, were generally higher than with FA. In particular the ACM statistics of the right posterior corona radiata, and the right superior longitudinal fasciculus showed a stronger correlation with the EDSS scores than the corresponding FA statistics (Figure 5).

**Table 4:** The results of ROI specific ACM/FA disease score correlations and GLM analyses.

Region of interest	Pearson's correlation		GLM with correction for age and gender		r^2^	
	ACM	FA	ACM	FA	ACM	FA
R-superior longitudinal fasciculus	−0.46(b*)	NS	0.57(b*)	0.51(b*)	0.33	0.26
R-corticospinal tract	−0.29(*)	NS	0.50(b*)	0.50(b*)	0.25	0.25
R-Posterior corona radiate	−0.62(b*)	NS	0.70(b*)	0.53(b*)	0.49	0.28
L-Posterior corona radiate	NS	NS	0.52(b*)	0.53(*)	0.27	0.28

**Legend:** The first column lists of a WM region of interest delineated in the JHU-ICBM-DTI-81 atlas with the letters R and L indicating a specific hemisphere. The second and third columns show Pearson's correlations between the summary statistics of median ACM or median FA and the EDSS disease scores. Boneferroni correction is used to account for multiple ROI comparisons (n = 10) and the (b*) indicates a Boneferroni corrected significant correlation at a significance level of 0.05 and (*) indicates uncorrected significance (0.05 level) while NS means not significant. The fourth and fifth column gives the linear estimate of disease score prediction based on a general linear model (GLM) including covariates of age, gender and either the median ACM or median FA while r^2^ is the coefficient of determination, summarizing the percentage of variance explained by the model.

For completeness and in comparison with Table 4, we note that Pearson's linear correlation coefficient between the skull-normalized lesion load and the EDSS was 0.316 (p-value<0.05), while the GLM adjusted for age and gender had fitting correlation 0.59 (p-value<0.05). The coefficient of determination value was r^2^ = 0.35.

## Discussion

Based on the connectivity information of ACM, we found widely reduced anatomical connectivity in the motor-related WM of RR-MS and SP-MS patients compared to healthy subjects. SP-MS patients showed voxel-wise reduced ACM values relative to RR-MS while no consistent decreases were found using FA. Accordingly, the motor-related disease symptoms of the two MS groups are significantly different; however the difference is only measurable using ACM. Regional ACM summary statistics correlated moderately with clinical disability as reflected by the EDSS score and showed correlation similar to or higher than FA summary statistics.

### 4.1. Reduced connectivity in MS patients

Voxel-based analyses and region specific statistics provided converging evidence for a reduction in the cerebral WM for the ACM of MS patients, compared to a group of healthy subjects. This is in contrast with a previous study where ACM has been investigated in patients with RR-MS [14]. In that study, ACM changes were found in the subcortical GM nuclei of the thalami and caudate nuclei but not in WM. We have shown that MS is also associated with a widespread change of anatomical connectivity in motor-related WM tracts. This decreased connectivity was found in both the RR-MS and SP-MS patients. We believe the discrepancies related to previous work of [14] are largely attributed to differences in how we estimate and analyse the ACM relative to how they do it. In [14] an ACM estimation was used which can potentially cause the ACM values to be related with the size of brain structures. While such brain structure size differences may in themselves be relevant to pathological processes our ACM estimation takes place in a spatially normalized which minimizes such structural size dependence relation and therefore may provide a clearer assessment of connectivity-related differences.

Several pathological processes may have contributed to reducing the ACM in patients with MS. Axonal damage, Wallerian degeneration, local inflammation and demyelination, all contribute to changing the ACM values, since these pathologies may affect the diffusion process, leading to changes in the principal directional uncertainty of the fODF, propagating through to the ACM. The decreased ACM of MS patients widely summarizes these pathologies, since an ACM voxel obtains its value based on streamlines that will have passed through areas of the WM tract with potentially different underlying disease pathology. Consequently an ACM not only reflect the localized tissue pathology as with DTI indices. It also reflects the accumulated effect that pathologies have on estimating a structural WM connection from a given voxel. As may be expected the results show decreased connectivity in patients with more severe symptoms.

In addition to voxel-wise ACM reductions, MS patients also exhibited decreases in FA, suggesting that both ACM and FA are sensitive to measuring MS-related changes, although they have different interpretation. As with ACM values, FA changes can be a function of axonal damage, Wallerian degeneration, local inflammation and demyelination. However, the interpretations of the measurements are quite different. In particular the values of an ACM measure a spatially dependent whole-brain effect derived from the trajectories of streamlines passing through multiple voxels. In distinction, FA measures local microstructural tissue changes, which may to some degree also reflect distant tissue damage. While there may be some correlation between ACM and FA, they are by design sensitive to very different aspects of the dMRI measurements. The FA decreases observed here agree with the findings of [11] reporting widely decreased FA within the major WM tracts of MS patients. Comparison of the regions of significantly decreased voxels with those of the probabilistic lesion map revealed a minimum overlap with the ACM findings while the overlap was much larger with FA. This suggests that the ACM is less sensitive to measuring highly localized WM damage compared to FA.

### 4.2. Decreased connectivity in SP-MS relative to RR-MS

We believe the difference between ACM and FA explains why group-wise comparisons based on ACM resulted in significant differences between SP-MS and RR-MS patients, while we were unable to find differences using FA. Additionally, the median ACM statistic of the entire motor-related WM ROI was significantly lower in patients with SP-MS than in patients with RR-MS. Together; these findings highlight the potential use of ACM to capture alterations in the connectivity pattern which are characteristic to the different clinical forms of MS.

The significant voxel-based ACM decreases found was hypothesized prior to undertaking the study. The strongly localized ACM reductions in patients with SP-MS are plausible as the patient group with RR-MS had less motor impairment as reflected by their EDSS scores. The ACM decreases of SP-MS found in the superior longitudinal fasciculus were hypothesized to be motor-related but could also be related to differences in distinct cognitive functions between both groups. Since we did not expose patients to cognitive tests, we cannot comment on this possibility.

Supplementing these findings, the differences in lesion probability maps between the two phenotypes were found to be non-significant. This is consistent with the finding of very few phenotype-related FA-related differences but in stark contrast with ACM. The ACM-related differences suggest it is indeed able to capture/quantify system specific spatially complex pathologies; in a way not observable using FA or lesion probability maps.

### 4.3. Correlation between ACM and clinical disability

In our patient cohort, region-specific median ACM statistics calculated within the WM ROIs correlated with MS disability as indicated by the EDSS score. Negative correlations were found between the median ACM statistics and individual EDSS scores. In contrast to our results, the study by [14] found no voxel-based correlation between ACM and the EDSS in patients with RR-MS. Several factors may have attributed to these negative findings. For instance the fact that they examined voxel-based correlations as opposed to ROI-based, the smaller range of inter-subject variation in EDSS of their cohort compared with ours and differences in cohort sample sizes. There are also large methodological differences in the ACM estimation which could cause differences between the results.

Correlation analyses did not reveal any significant correlations between the median FA statistics and individual EDSS. However, when applying a GLM that adjusted for between-subject differences of age and gender, we found a linear relationship with EDSS for both the median ACM and median FA. The explanatory power of the linear relation was generally stronger for using the ACM than it was using FA. The median ACM in the right posterior corona radiata, right superior longitudinal fasciculus correlated more strongly with the EDSS than the corresponding median FA. Our results confirm that ACM is at least as good a marker of MS-related disability as FA. Since ACM and FA provide complementary information, it is possible that a combined measure that integrates the structural information provided by FA and ACM might be most sensitive to disability and disease progression. This possibility remains to be addressed in depth in future studies.

The findings suggest that ACM may be used to relate regional anatomic disconnection with disease-related disabilities. A similar finding was made in a study involving amyotrophic lateral sclerosis patients, in which a tract-specific probabilistic index of the corticospinal tract was found to achieve better disease score correlation than FA [45] and our analysis suggest a similar trend. Accordingly [14], demonstrated that ACM values in the anterior body of the corpus callosum correlated with the number of correct responses in the Pace Auditory Serial Addition Test (PASAT) in patients with RR-MS. Together, these findings suggest that ACM has the potential to unravel interesting relations between altered anatomical connectivity and disease-related disabilities. In this context, the use of specific motor or cognitive tests, for instance measurements of ankle dorsiflexion and hip flexion strength [37], the pyramidal functional system score of the EDSS score, the Ambulation Index but also the Multiple Sclerosis Functional Composite Score (MSFC), may be used to inform future ACM studies to find out how altered anatomical connectivity in specific tracts affect specific motor and cognitive functions.

### 4.4. Methodological considerations

When ACM is applied to patients with MS, probabilistic tractography needs to measure isotropic diffusion components in the affected brain tissue due to inflammation, demyelination, gliosis, and axonal injury. We combined the multi-tensor model with the Bingham distribution [46], used with the probabilistic tracking method of [29] in order to capture these effects.

The multi-tensor model is one of numerous models that could be used for reconstructing local fiber configurations of WM. In the context of ACM, [13]–[14] have previously used the Qball model [47] but in principle numerous models could be used some of which are described in the overview chapter of [48]. These authors list the advantages and disadvantages of different models. According to this comparison the Qball and multi-tensor model are on par with respect to resolving fibers in normal tissue. We chose the tensor model since it is relatively easy to spatially deform; a property we use to estimate the ACMs in a population wide common space, as opposed to individual space. The choice of model is however a critical one since it determines the ability to resolve fiber configurations and the subsequent accuracy of tractography. In MS this task is particularly challenging due to the complex MS pathology (inflammation, demyelination, gliosis, and axonal injury). The lack of knowledge about the impact MS pathology may have on the fiber reconstruction model is a generic weakness of current fiber reconstruction models and any such fiber reconstruction errors will have impacted the probabilistic tracking and possibly the resulting ACMs. Another issue that may impact the reliability of the ACMs is the number of streamlines used in its estimation. This is one of the methodological differences that may have caused discrepancy with respects to a previous ACM-based MS study of [14]. We used a conservatively large number of streamlines (500) for this study. Previous ACM studies have used just 10 streamlines per voxel [12]–[14] but our SNR investigations suggested that such a low number of streamlines may lead to a high uncertainty of the ACM connectivity estimates. Another important difference relative to previous works using ACM is that we have used a spatially normalized ACM technique similar to [26]. Normalization ensured that the connectivity estimates of ACM become minimally dependent on the individuals brain shape and size. As part of performing the voxel-based study, the ACM data were smoothed using a Gaussian kernel with a full-width half-maximum value of 4 mm. This value is at the low end of what many voxel-based DTI studies employ [31]. It was chosen by comparing the results of using 4 mm and 8 mm and observing only a minor difference in the SVC results. Since the full-width half-maximum have previously been interpreted as a prior knowledge [31], [49] deciding the spatial extent of the potential detectable effects we choose the value of 4 mm favouring results corresponding to the least prior assumption. This choice is subject to the assumption that the ACMs contain a minimal amount of noise due spatial normalization errors.

## Conclusion

We introduced a method for obtaining a spatially normalized ACM, suited for group studies to account for the integrated pathology occurring along WM tracts of MS patients. Unlike previous work, this way of estimating ACM ensured that the connectivity estimates of ACM become minimally dependent on the individuals brain shape and size variation. We successfully applied the ACM, to detect consistent differences in whole-brain anatomical connectivity between two clinical sub-groups of MS. We were able to relate alterations in the ACM with disease-related disability in motor-related WM and showed that ACM provides complementary information to conventional DTI indices such as FA. However further large scale studies are warranted to explore the potential of ACM to map the magnitude and spatial distribution of anatomical disconnection in brain diseases and relate these connectivity changes with disease-related disabilities.
